# Prevalence and genetic characterization of *Toxoplasma gondii* in naturally infected backyard pigs intended for familial consumption in Romania

**DOI:** 10.1186/s13071-019-3842-8

**Published:** 2019-12-16

**Authors:** Anamaria Ioana Paştiu, Anamaria Cozma-Petruț, Aurélien Mercier, Anamaria Balea, Lokman Galal, Viorica Mircean, Dana Liana Pusta, Liviu Bogdan, Adriana Györke

**Affiliations:** 1 Department of Parasitology and Parasitic Diseases, Faculty of Veterinary Medicine, University of Agricultural Sciences and Veterinary Medicine Cluj-Napoca, 3-5 Calea Mănăştur Street, 400372 Cluj-Napoca, Cluj Romania; 2 Department of Genetics and Hereditary Diseases, Faculty of Veterinary Medicine, University of Agricultural Sciences and Veterinary Medicine Cluj-Napoca, 3-5 Calea Mănăştur Street, 400372 Cluj-Napoca, Cluj Romania; 3 Department of Bromatology, Hygiene, Nutrition, Faculty of Pharmacy, “Iuliu Haţieganu” University of Medicine and Pharmacy, 6 Pasteur Street, 400349 Cluj-Napoca, Cluj Romania; 4 INSERM UMR_S 1094, Neuroépidémiologie Tropicale, Laboratoire de Parasitologie-Mycologie, Faculté de Médecine, Université de Limoges, Limoges, France; 5 Centre National de Référence Toxoplasmose/Toxoplasma Biological Resource Center, CHU Limoges, 2 Martin Luther King Street, 87042 Limoges, France; 6 Department of Reproduction, Obstetrics and Pathology of Animal Reproduction, Faculty of Veterinary Medicine, University of Agricultural Sciences and Veterinary Medicine, 3-5 Calea Mănăştur Street, 400372 Cluj-Napoca, Cluj Romania

**Keywords:** *Toxoplasma gondii*, Pigs, Prevalence, Genotyping, Romania

## Abstract

**Background:**

Foodborne toxoplasmosis in humans can be due to the exposure to tissue cysts of *Toxoplasma gondii* through the consumption of meat, including pork, of infected animals. Traditional Romanian food habits include pork as the preferred meat, while backyard pig rearing remains a common practice in many rural areas of Romania. The aims of the present study were to estimate the prevalence of *T. gondii* infection in naturally infected backyard pigs slaughtered for familial consumption and to genetically characterize the *T. gondii* strains obtained.

**Methods:**

Paired blood and heart samples were collected from 94 backyard pigs, home slaughtered for private consumption. Serum samples were analyzed using the immunofluorescence antibody test (IFAT) for anti-*T. gondii* antibody detection. Heart samples were screened by polymerase chain reaction (PCR) targeting the 529-bp repeat region (REP529) for *T. gondii* detection. In addition, heart samples from IFAT positive animals were bioassayed in mice. The *T. gondii* isolates were genotyped by the analysis of 15 microsatellite markers.

**Results:**

The results showed that almost half of the pigs investigated were *T. gondii* seropositive (46.8%, 95% confidence interval (CI): 36.4–57.4%) and in more than a quarter of the pigs (26.6%, 95% CI: 18.0–36.7%), the parasite was detected by PCR. Three (3/44) *T. gondii* strains were isolated from hearts of seropositive pigs and they all belonged to genotype II.

**Conclusions:**

The present study showed the presence of *T. gondii* infection in backyard pigs in Romania, which suggests that consumption of pork from animals reared and slaughtered at home may pose a potential threat to human health and should be given attention. In addition, to our knowledge, this is the first study to provide data concerning *T. gondii* strains circulating in pigs from Romania.

## Background

*Toxoplasma gondii*, a coccidian parasite of the family Sarcocystidae, is one of the most studied parasites because of its medical and veterinary importance. This parasite induces parasitic infection in humans and other warm-blooded animals [1]. Backyard rearing of livestock represents an important source of food, but also contributes to human exposure to different zoonotic pathogens. Pigs are one of the species in which *T. gondii* is often found [2, 3]. Worldwide, the prevalence of anti-*T. gondii* antibodies in pigs was estimated to be 19%, with Europe recording the lowest values and Africa and North America having a high prevalence, respectively [3]. *Toxoplasma gondii* prevalence in pigs can vary according the age of animal and the animal husbandry system, backyard pigs being more exposed to the parasite compared to indoor pigs [4]. Likewise, the higher mean annual temperature and lower geographical latitude were identified as risk factors for *T. gondii* infection [3].

Regarding the possibility of infection with *T. gondii*, it should be taken into account that meat processing methods have an important role in the viability and infectivity of *T. gondii* tissue cysts. In order to inactivate *T. gondii* in meat, it is recommended to cook whole cuts of pork to an internal temperature of at least 65.6 °C, with a three-minute rest [5]. Infectivity of *T. gondii* cysts is also influenced by processing methods such as curing [6] or freezing [7] as well as by the interaction between salt concentration, maturation time and temperature [8].

It is estimated that up to one third of human population worldwide is *T. gondii* seropositive [9]. In Romania, a higher *T. gondii* seroprevalence was reported in individuals from rural areas (76.9%) when compared to those from urban regions (55.3%) [10]. In the countries with a temperate climate, between 30% and 63% of infections have been attributed to the consumption of undercooked or cured meat products, whereas only 6% to 17% to soil contact [11, 12]. Moreover, there were reported cases of toxoplasmosis related to the consumption of pork or cured pork products in Italy [13, 14], the USA [15] and Korea [16].

The traditional Romanian food habits are based on meat. The annual average meat consumption per capita is around 65 kg [17]. Pork is the preferred meat in Romania, covering almost half of the meat consumption, and its consumption is considerably increasing during winter, especially during Christmas. In the food habits of the local population, especially from the north-west of Romania, the pork products are consumed raw, processed only by smoking and/or salting [18]. Most pork may originate from two sources: backyard pigs, with 1 to 3 pigs per unit, raised for familial consumption and slaughtered at home at the age of 10–12 months (100–120 kg); and indoor pigs, raised for large consumption, slaughtered in abattoirs at the age of 6–8 months (80–90 kg) [19].

However, backyard rearing of pigs, together with other animals such as cats, dogs, chickens etc., remains a common practice in many Romanian rural areas [4]. So far, only a few studies have focused on *T. gondii* infection in this type of pig production system in Romania [4]. Likewise, to our knowledge, no information on the genetic characterization of *T. gondii* isolates from pigs in Romania is available. Thus, the aims of this study were to estimate the seroprevalence of *T. gondii* antibodies and to genetically characterize the *T. gondii* strains obtained from backyard pigs intended for familial consumption.

## Methods

### Study area and sample collection

**Fig. 1:**
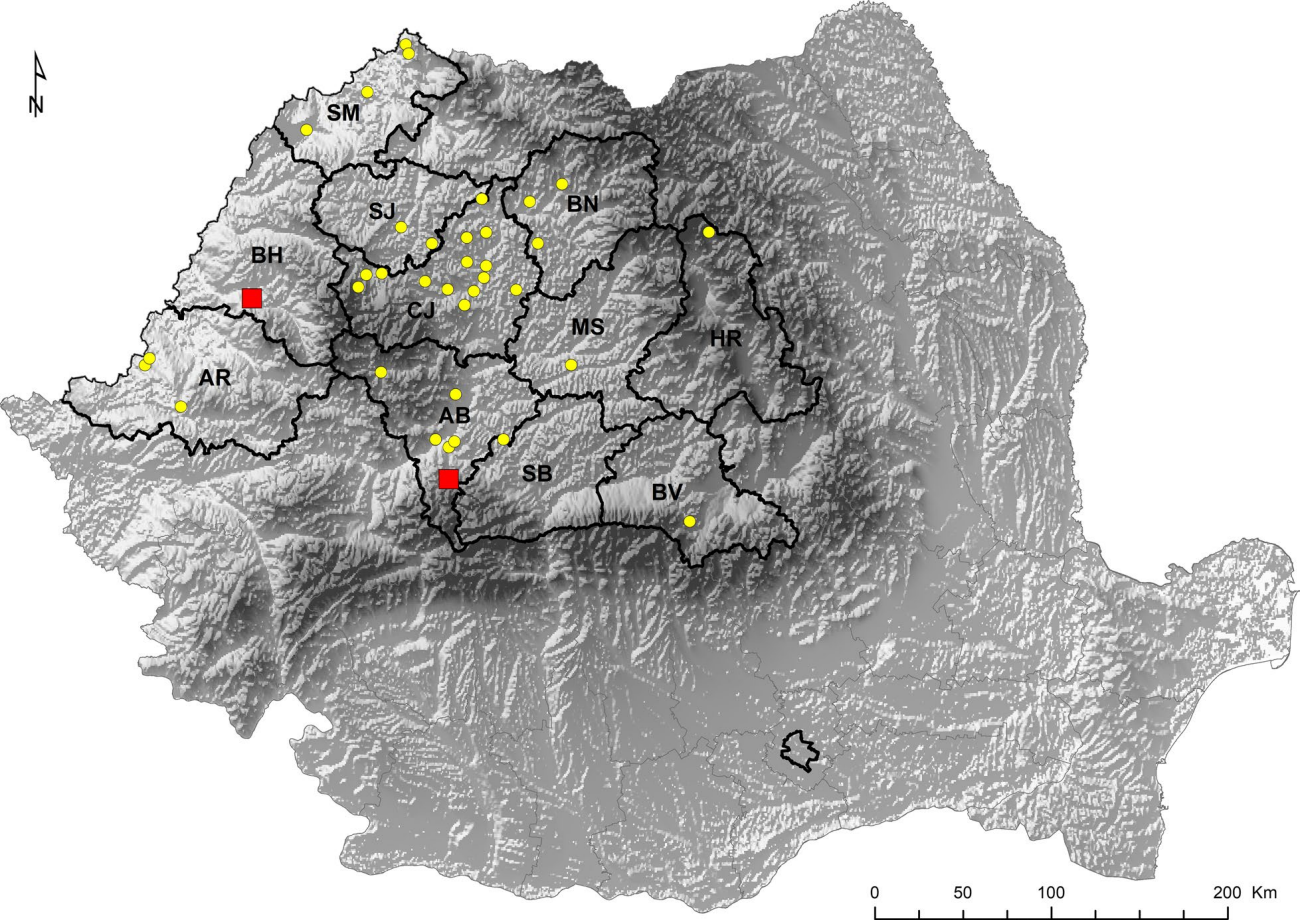
Regional distribution over Romania of households with backyard pigs included in the study. Yellow circles, sites where blood and heart samples were collected from backyard pigs; red squares, sites where *T. gondii* isolates were obtained. The map was created with ArcGis software. *Abbreviations*: AB, Alba county; AR, Arad county; BH, Bihor county; BN, Bistrița-Năsăud county; BV, Brașov county; CJ, Cluj county; HR, Harghita county; MS, Mureș county; SB, Sibiu county; SJ, Sălaj county; SM, Satu Mare county

From December 2011 to March 2013, paired samples of blood and heart (*n* = 194) were collected randomly from backyard pigs. The samples were obtained in 67 different backyard holdings, from 11 counties in central, north-western and western Romania (Transylvania) (Fig. 1). The backyard pigs were reared in small extensive farms, 1–3 pigs per unit, were slaughtered at home and were used for familial consumption. The pig owners voluntarily participated in the study. A questionnaire was prepared for collection of data regarding the age, sex and region of origin of the animals. From each pig, a blood sample was collected from the jugular vein during the slaughtering. Heart samples were collected individually. The samples were transported under refrigerated conditions to the laboratory. Serum samples were tested by IFAT within 24–48 h. Heart tissue samples were kept under refrigeration conditions (4 °C) until bioassay in mice was performed. Only heart samples from seropositive animals were selected for bioassay in order to isolate and genotype the *T. gondii* strains.

### Immunofluorescence antibody test (IFAT)

Serum samples were analyzed by an in-house IFAT assay for anti-*Toxoplasma* specific IgG-antibodies as previously described by Györke et al. [20], using a cut-off titer of 32. Two dilutions were performed 1:32 and 1:64. Whole tachyzoites of *T. gondii* RH strain were used as antigen (in-house product of the Laboratory of Parasitology, Faculty of Veterinary Medicine, UASVM-CN, Romania). Positive and negative controls were used from a previous epidemiological study [4].

### Bioassay

The heart samples from pigs identified as seropositive by IFAT were bioassayed on pathogens-free Swiss mice, in order to isolate the viable *T. gondii* strains. The protocol described in the study of Paştiu et al. [21] was used. Briefly, each heart sample (8–95 g) was minced and mixed (Knife Mill Grindomix GM 200, Retsch, Haan, Germany) with digestion solution (0.25% trypsin (93613, Sigma-Aldrich, Saint-Louis, USA)/0.025% EDTA). The homogenate was incubated for 1.5 h at 37 °C and then filtered through gauze. The suspension was centrifuged at 1800×*g* for 10 min. The supernatant was discarded, and the pellet was washed 3 times with phosphate buffered saline (PBS, pH 7.2) by centrifugation. As a last step, the pellet (digest) was mixed with 3 ml PBS containing 100 µl antibiotic solution (20,000 units penicillin/10,000 units streptomycin, P0781, Sigma-Aldrich) [22]. For each sample, 0.5 ml of digest was inoculated intraperitoneally in two Swiss mice.

The mice were monitored for clinical signs of toxoplasmosis, twice daily, for 4 weeks, then euthanized and their brains recovered. The mice were euthanized earlier if signs of acute toxoplasmosis (ruffled fur, diminished response to handling, state of prostration) were observed for three consecutive days. Mouse brains were mechanically homogenized and examined microscopically between a glass slide and coverslip using ×10–40 magnifications. For each brain, 5 glass slides were prepared. Also, the mouse brains were analyzed by polymerase chain reaction (PCR) [23].

### *Toxoplasma gondii* DNA detection

DNA was extracted from heart samples and from the mice brains (40 mg/sample) using a commercial kit (Isolate Genomic DNA Kit, Bioline, London, UK) according to the manufacturer’s protocol. Positive control was represented by DNA obtained from *T. gondii* RH strains, but no negative DNA extraction controls were used. DNA was amplified using the 529-bp DNA fragment [23] as previously described by Paştiu et al. [24]. Positive and negative controls were included in each run. A negative control was represented by distilled ultrapure water. No internal control was included in the PCR.

### Genotyping analysis

*Toxoplasma gondii-*positive DNA samples which were extracted from the mouse brain homogenate were submitted for genotyping using 15 microsatellite markers (N61, B18, M33, M48, TUB2, N83, XI.1, N82, TgM-A, W35, IV.1, B17, N60, M102 and AA) distributed on 11 of the 14 chromosomes that comprise the *T. gondii* genome, as described elsewhere [25]. Briefly, the forward primer from each pair was 5′-end labelled with fluorescein as follow: 6-carboxyfluorescein (6-FAM) was used for TUB2, XI.1, B18, N83, N61, M33 and M48; hexachlorofluorescein (HEX) for MS TgM-A, B17, N82, W35 and IV.1; and 2,7′,8-benzo-5′-fluoro-2,4,7-trichloro-5-carboxyfluorescein (NED) for AA, N60 and M102. The PCR was carried out in a 25 µl reaction mixture consisting of 12.5 µl of 2× Qiagen Multiplex PCR Master Mix (Qiagen, Courtaboeuf, France), 5 pmol of each primer and 5 µl DNA. Cycling conditions were: initial denaturation 15 min at 95 °C, followed by 35 cycles of 94 °C for 30 s, 61 °C for 3 min, 72 °C for 30 s, and 30 min at 60 °C. PCR products were diluted 1:10 with deionized formamide (Applied Biosystems, Life Technologies, Carlsbad, California). One microliter of each diluted PCR product was mixed with 0.5 µl of a dye-labelled size standard (ROX 500, Applied Biosystems) and 23.5 µl of deionized formamide (Applied Biosystems). This mixture was denatured at 95 °C for 5 min. The PCR products were electrophoresed using an automatic sequencer (ABI PRISM 3130xl, Applied Biosystems). The size of the alleles in bp was estimated using GeneMapper analysis software (version 4.0, Applied Biosystems).

An unrooted neighbour-joining dendrogram was produced from the microsatellite data using Populations 1.2.32 (http://bioinformatics.org/populations/) based on Cavalli-Sforza and Edwards [26] chord distance estimator and generated with MEGA7 (http://www.megasoftware.net/history.php).

### Statistical analyses

Frequency, apparent prevalence and its 95% confidence interval (95% CI) were calculated for the anti-*T. gondii* antibodies and *T. gondii* DNA. These parameters were determined overall and by age group (young, < 1 year old; adults, ≥ 1 year old), sex (males, females), and for each region (central, north-western, western). The difference in prevalence among groups was statistically analyzed using a Chi-square test of independence. A *P*-value of < 0.05 was considered statistically significant. Data were processed using EpiInfo 2000 software (CDC, Atlanta, GA, USA).

In addition, the level of agreement between *T. gondii* detection methods (IFAT and PCR) was calculated using the overall agreement measure and Cohen’s Kappa statistic with the Win Episcope 2.0 program. Positive and negative percent agreement was also calculated. Interpretation of *k* index was performed as follows: < 0, no agreement; 0–0.20, slight agreement; 0.21–0.40, poor agreement; 0.41–0.60, moderate agreement; 0.61–0.80, good agreement; and 0.81–1, very good agreement [27].

## Results

Forty-four out of 94 pigs (46.8%, 95% CI: 36.4–57.4%) were seropositive for *T. gondii,* with a titer of 1:32 (as a cut-off point). The household seroprevalence level was 49.25% (33/67). A similar exposure to *T. gondii* infection was observed, the pigs originated from the same household being all seropositive or seronegative, respectively. The prevalence was significantly higher in the central area (56.8%, *χ*^2^ = 7.2648, *df* = 2, *P* = 0.04) than in the western area (10%). There were no statistically significant differences in *T. gondii* seroprevalence between males (53.3%) and females (40.8%) (*χ*^2^ = 1.4761, *df* = 1, *P* = 0.22), and between adult (50.9%) and young pigs (40.5%) (*χ*^2^ = 0.9628, *df* = 1, *P* = 0.33) (Table 1). The endpoint serum dilution was 1:32 for 28.7% of samples and 1:64 for 18.1% of samples, respectively.

**Table 1:** Prevalence levels of *T. gondii* infection in backyard pigs from Romania by region, age and sex using IFAT and PCR

		IFAT			PCR		
	*n*	No. positive	Prevalence (95% CI)	*P*-value	No. positive	Prevalence (%) (95% CI)	*P*-value
Region							
Central	44	25	56.8 (41.0–71.7)*	0.04	7	15.9 (6.6–30.1)	0.04
North-west	40	18	45.0 (29.3–61.5)		14	35.0 (20.6–51.7)*	
West	10	1	10.0 (0.25–44.5)		4	40 (12.2–73.7)	
Age group							
< 1 year-old	37	15	40.5 (24.8–57.9)	0.33	10	27.0 (13.8–44.1)	0.79
≥ 1 year-old	57	29	50.9 (37.3–64.4)		14	24.6 (14.1–37.8)	
Sex							
Females	49	20	40.8 (27.0–55.8)	0.22	11	22.4 (11.8–36.6)	0.47
Males	24	45	53.3 (37.9–68.3)		13	28.9 (16.4–44.3)	
Total	94	44	46.8 (36.4–57.4)		24	25.5 (17.1– 35.6)	

*P < 0.05

*Abbreviations*: CI, confidence interval; IFAT, immunofluorescence antibody test; n, number of samples; PCR, polymerase chain reaction

*Toxoplasma gondii* DNA was found in 25 out of 94 heart tissues (26.6%, 95% CI: 18.0–36.7%). There were no statistically significant differences in *T. gondii* prevalence between regions (*χ*^2^ = 4.9415, *df* = 2, *P* = 0.08), between males (28.9%) and females (22.4%) (*χ*^2^ = 0.5117, *df* = 1, *P* = 0.47), and between adult (27%) and young pigs (24.6%) (*χ*^2^ = 0.0717, *df* = 1, *P* = 0.79) (Table 1). The negative controls were always negative.

IFAT (46.8%) has shown a higher prevalence of *T. gondii* infection in pigs compared to PCR (25.5%) (Table 1), but without statistically significant differences (*P* > 0.5). Overall, 57.4% (*n* = 54) of pigs were positive at least in one of the performed tests. Discordant results were found in 40 cases (42.6%): 30 (31.9%) samples were positive only in IFAT and 10 (10.6%) samples were positive only for *T. gondii* DNA. Cohen’s Kappa statistic showed a slight agreement between indirect (IFAT) and direct (PCR) methods (*k* = 0.12) (Table 2). The overall agreement was 57.7%, the positive agreement 41.2% and the negative agreement 66.7%, respectively (Table 2).

**Table 2:** Measures of agreement between IFAT and PCR results

	IFAT/PCR heart
Kappa (*k*)	0.12 (− 0.06–0.30)
Overall agreement (%)	57.5
Positive percent agreement	41.2
Negative percent agreement	66.7
McNemar	9.03

*Abbreviations*: IFAT, immunofluorescence antibody test; PCR, polymerase chain reaction

**Fig. 2:**
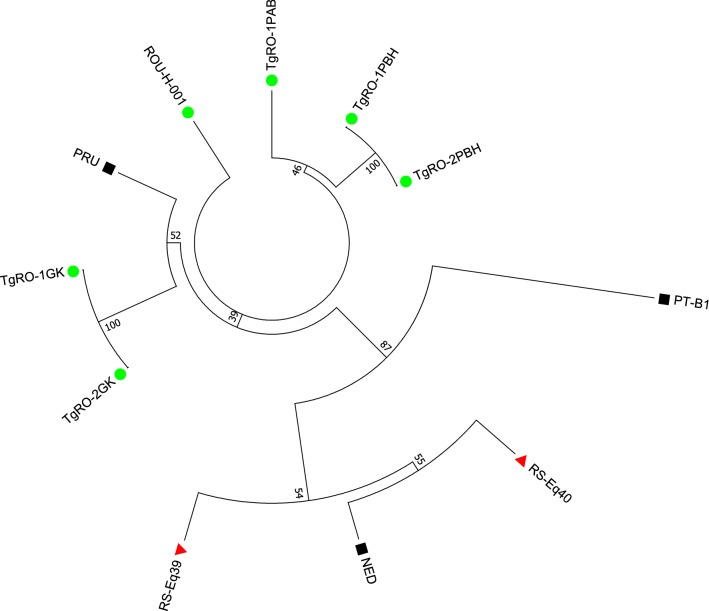
Neighbour-joining clustering of *T. gondii* isolates based on 15 microsatellite markers. Green circles next to the identifiers of genotypes indicate strains from Romania (*n* = 16), red triangles indicate strains from the neighbouring Serbia (*n* = 12) [47] and black squares indicate reference strains representing the three most common lineages in Europe: types I, II and III. Support values using 1000 bootstrap samples are shown at the base of each clade. For details refer to Table 3

*Toxoplasma gondii* was isolated by mice bioassay from 3 out of 44 seropositive pigs (6.82%, 95% CI: 1.4–18.7%). Also, these pigs were positive in IFAT and PCR from heart tissues. PCRs performed on DNA extracted from mouse brains confirmed the results obtained in the bioassay. The isolates were obtained from pigs sampled in Bihor (*n* = 2) and Alba (*n* = 1) counties. The two sampling sites are located at a distance of 224 km. The *T. gondii* strains were designated as TgRO-1PBH, TgRO-2PBH and TgRO-3PAB, respectively. All three isolates were obtained from males, aged ≥ 1 year-old. The *T. gondii* isolates had a type II genotype (Table 3, Fig. 2). Two of them, TgRO-1PBH, TgRO-2PBH, were genetically identical.

**Table 3:** Genotyping results of *Toxoplasma gondii* DNA from Romanian strains (*n* = 13) isolated from pigs and a set of Romanian and European strains isolated from animals and humans and analyzed in previous studies with 15 microsatellite markers in a single multiplex PCR assay

Isolate	Origin	Host species	Lineage	Microsatellite markers															
				TUB2	W35	TgM-A	B18	B17	M33	IV.1	XI.1	M48	M102	N60	N82	AA	N61	N83	Ref
PT-B1	Portugal	Cow	Type I	291	248	209	160	342	169	274	358	209	166	147	119	273	87	306	[48]
PRU	France	Human	Type II	289	242	207	158	336	169	274	356	209	176	142	117	265	123	310	[49]
NED	France	Human	Type III	289	242	205	160	336	165	278	356	209	190	147	111	267	91	312	[25]
ROU-H-001	Romania	Human	Type II	289	242	207	158	336	169	274	356	231	176	138	109	273	93	312	[46]
TgRO-1GK	Romania	Goat	Type II	289	242	207	158	336	169	274	356	235	176	140	115	275	115	310	[21]
TgRO-2GK	Romania	Goat	Type II	289	242	207	158	336	169	274	356	235	176	140	115	275	115	310	[21]
RS-Eq39	Serbia	Horse	Type III	289	242	205	160	336	165	278	356	211	190	147	111	265	87	312	[47]
RS-Eq40	Serbia	Horse	Type III	289	242	205	160	336	165	278	356	213	190	149	111	267	89	312	[47]
TgRO-1PAB	Romania	Pig	Type II	289	242	207	158	336	169	274	356	225	176	142	113	259	95	312	This study
TgRO-1PBH	Romania	Pig	Type II	289	242	207	158	336	169	274	356	217	176	142	125	277	93	312	This study
TgRO-2PBH	Romania	Pig	Type II	289	242	207	158	336	169	274	356	217	176	142	125	277	93	312	This study

*Abbreviation*: Ref, reference

## Discussion

The seroprevalence of anti-*T. gondii* antibodies in backyard pigs from central, north-western and western Romania was 46.8% by IFAT. In a previous comparative assessment of *T. gondii* seroprevalence in pigs using IFAT, modified agglutination test (MAT) and enzyme-linked immunosorbent assay (ELISA), IFAT obtained the best sensitivity (97.4%, 95% CI: 92.3–100%) and specificity (96.4%, 95% CI: 91.6–100%) [28]. Surveys based on the presence of anti-*T. gondii* antibodies in pig’s serum samples, using different detection methods, especially ELISA, MAT and IFAT, have been reported worldwide [1]. In Europe, the *T. gondii* seroprevalence has been estimated to be 13% (95% CI: 10–15%) [3]. Few data are available on *T. gondii* seroprevalence in pigs in eastern Europe. A seroprevalence of 30.5% was reported in backyard pigs in Romania [4], similar studies from eastern Europe report lower values such as 5.8% in Estonia [29], 17% in Serbia [30], 19.2% in Poland [31] and 27.6% in the Czech Republic, respectively [32]. Considering that the samples in the present study were collected from 2011–2013, further research is required to assess the current seroprevalence of *T. gondii* in backyard pigs in Romania.

In the present study, almost half of the tested backyard pigs were positive for IFAT, while a quarter were positive for PCR. The small quantity of heart tissue (40 mg) used for DNA extraction, as well as an inhomogeneous distribution of *T. gondii* cysts in tissue samples [33] often decreases the sensitivity of PCR-based methods and can further explain the difference between serology and *T. gondii* DNA detection [30, 34]. There are other PCR techniques (MC-qPCR), which are more sensitive than conventional PCR [35]. In contrast, negative serology together with a *T. gondii* DNA-positive result can be due to the low sensitivity of the method used for antibody detection [36]. Moreover, contamination or PCR inhibition can be potential reasons for a low concordance between PCR and serology methods. However, all these results demonstrate the presence of *T. gondii* without providing data on the infectivity and viability of the parasite.

The gold standard method for *T. gondii* detection is represented by bioassay in mice or cats [1]. Considering the low parasite load in the tissues of most *T. gondii* hosts, mouse bioassay is required to increase the parasite burden [37]. The size of samples bioassayed in mice can influence the results. In the present study, the three *T. gondii* isolates (TgRO-1PBH, TgRO-2PBH and TgRO-1PAB) were obtained from samples sized between 63.72–80.15 g. The results were in agreement with other studies, which recommended between 50–100 g of tissue for bioassay in mice [22, 33]. The low number of *T. gondii* isolates obtained may be due to the variation in size of heart samples and to the fact that only seropositive samples were tested by bioassay.

In the present study, *T. gondii* strains isolated from backyard pigs belonged to the type II genotype. Two of the isolates shared precisely the same alleles at all the 15 microsatellite loci. Considering the very high discrimination power of multilocus genotyping with microsatellite markers [25], the result obtained suggests that TgRO-1PBH and TgRO-2PBH are identical and belong to a single strain. Moreover, the two *T. gondii* strains were isolated from pigs from the same household. The third isolate, named TgRO-1PAB, showed minor multilocus variation at 2 of the 15 microsatellite markers, in comparison with the two first genotypes (Fig. 2). To our knowledge, this is the first study in Romania to report the isolation of viable *T. gondii* from seropositive pigs reared in an extensive growth system.

*Toxoplasma gondii* type II, followed by type III are the most prevalent genotypes detected in Europe [38–41], but few data are available on *T. gondii* genotypes in pigs in eastern Europe. In pigs, type II has previously been detected in Switzerland, Slovakia, Portugal, France, the Czech Republic, and Italy [32, 36, 42–45], while in Serbia both type II and III were reported [30].

Generally, in Romanian households, 1–3 backyard pigs are reared together with other animal species (e.g. cats, dogs, poultry and possibly rodents). The pigs are fed mainly kitchen leftovers and, in some households, they have outdoor access. The backyard pigs are slaughtered at home and used for consumption in the family [4]. Breeding backyard pigs is a necessity for many families in Romania, but it is important that education on the development of sanitary and animal health standards is undertaken. An increase in biosecurity measures and the development of health education in order to limit the spread of *T. gondii* infection are required in the country.

New information regarding the *T. gondii* strains present in backyard pigs in Romania are provided, which also helps complete the scarce data regarding *T. gondii* genotypic diversity in eastern Europe. To date, in Romania, previous studies have shown the presence of type II isolates obtained from humans [46] and goat-kids [21]. Further studies are required to develop a more complete picture of the *T. gondii* genotypes present in various species of domestic and wild animals in Romania.

## Conclusions

The present study reports the presence of *T. gondii* antibodies in backyard pigs from Romania. In addition, we also describe the presence of viable *T. gondii* from these seropositive pigs. This highlights the real risk of human contamination by consumption of raw/undercooked pork from backyard pigs in Romania.

## Data Availability

The data supporting the results and conclusions of this article are included within the article.

## Abbreviations

CI: confidence interval
ELISA: enzyme-linked immunosorbent assay
6-FAM: 6-carboxyfluorescein
HEX: hexachlorofluorescein
IFAT: immunofluorescence antibody test
MAT: modified agglutination test
MC-qPCR: magnetic capture-qPCR
NED: 2,7′,8′-benzo-5′-fluoro-2′,4,7-trichloro-5-carboxyfluorescein
PCR: polymerase chain reaction
